# Bisretinoid degradation and reduction of lipofuscin accumulation in the amelanotic retinal pigment epithelium of mammals with a tapetum

**DOI:** 10.1073/pnas.2315421120

**Published:** 2023-10-23

**Authors:** Bruce A. Pfeffer

**Affiliations:** ^a^Department of Ophthalmology, Jacobs School of Medicine and Biomedical Sciences, The State University of New York-University at Buffalo, Buffalo, NY 14215

In PNAS, Lyu et al. showed that melanin in the retinal pigmented epithelium (RPE) could be modified endogenously by reactive nitrogen species-induced chemiexcitation, generating high-energy triplet carbonyl functionality capable of degrading bisretinoids (naturally occurring products of the visual phototransduction process), including the signature molecule A2E (1). These reactions required melanolipofuscin derived from RPE phagolysosomes, a component of the photoreceptor outer segment renewal system, and did not result in cytotoxicity, in contradistinction to products formed by photodegradation (1). The authors’ contention that age-related macular degeneration (AMD) may result from the loss of melanosomes with advanced age was supported in an elegant fashion using a dioxetane precursor for triplet carbonyls, which reduced pathology in an albino mouse AMD model (1).

An exception to the above paradigm is by definition found in those mammals whose eyes feature a tapetum—a reflective element predominantly localized in the central aspect of the posterior segment, that increases photon capture in dim light—because in this region of the retina, the RPE is in fact amelanotic (“nonpigmented” RPE). Several different anatomical and functional manifestations of the tapetum occur across evolutionarily distinct taxa, varying with regard to the reflective substance, for example, in marsupials, bovids, carnivores, and Strepsirrhine prosimians (2, 3). There are no reports of accelerated lipofuscin accumulation, nor of retinal degenerations resembling AMD, in these species, even at the end of their natural lifespans. This suggests that pathways different from those involving melanin operate to prevent, moderate, and/or ameliorate the production, accumulation, and/or effects of lipofuscin, assuming that fundamental visual events already known to generate bisretinoids (4) are unchanged in the tapetal region of these retinas. Maintaining optical clarity dictates that mechanisms are in place in the tapetal retina-RPE interface to minimize or virtually eliminate interference from light-absorbing inclusions besides melanosomes, namely lipofuscin granules.

A previous report on circadian maxima for phagocytosis of shed disks from rods and cones in the tapetal retina of adult cats noted in passing the paucity of lipofuscin and also the timely disappearance of residual bodies (5). This raises the question of whether tapetal RPE cells are specialized for more efficient lysosomal degradation of phagosomes.

Various scenarios for mitigating lipofuscin accumulation have been proposed. Prelysosomal routes might include enhanced activity of Abca4, providing increased exposure of retinaldehyde to retinol dehydrogenase (6); taurine (an essential nutrient for cat retinal homeostasis) and other molecules acting as retinaldehyde-reactive sinks competing with phosphatidylethanolamine (7); delayed fusion of phagosomes with lysosomes (2, 5, 8); and modulation of interphotoreceptor retinoid-binding protein function (9).

Finally, tapetal RPE itself could implement a chemiexcitation-based mechanism for degrading bisretinoids. Here, in the absence of mature melanin, endogenously generated peroxynitrite would react with transparent melanin precursors and other indoles, yielding dioxetanes that resolve to triplet carbonyls (10). Proteins enriched in tryptophan (10) could be incorporated into lipofuscin granules in tapetal RPE for the disposal of bisretinoids and the maintenance of transmission of reflected light. Further study of tapetal RPE may have translational value for the prevention and treatment of AMD.
